# Anosmia and dysgeusia in SARS-CoV-2 infection: incidence and effects on COVID-19 severity and mortality, and the possible pathobiology mechanisms - a systematic review and meta-analysis

**DOI:** 10.12688/f1000research.28393.1

**Published:** 2021-01-21

**Authors:** Endang Mutiawati, Marhami Fahriani, Sukamto S. Mamada, Jonny Karunia Fajar, Andri Frediansyah, Helnida Anggun Maliga, Muhammad Ilmawan, Talha Bin Emran, Youdiil Ophinni, Ichsan Ichsan, Nasrul Musadir, Ali A. Rabaan, Kuldeep Dhama, Syahrul Syahrul, Firzan Nainu, Harapan Harapan

**Affiliations:** 1 Department of Neurology, School of Medicine, Universitas Syiah Kuala, Banda Aceh, Aceh, 23111, Indonesia; 2 Department of Neurology, Dr. Zainoel Abidin Hospital, Banda Aceh, Aceh, 23111, Indonesia; 3 Medical Research Unit, School of Medicine, Universitas Syiah Kuala, Banda Aceh, Aceh, 23111, Indonesia; 4 Faculty of Pharmacy, Hasanuddin University, Makassar, South Sulawesi, 90245, Indonesia; 5 Brawijaya Internal Medicine Research Center, Department of Internal Medicine, Faculty of Medicine, Universitas Brawijaya, Malang, 65145, East Java, Indonesia; 6 Research Division for Natural Product Technology (BPTBA), Indonesian Institute of Sciences (LIPI), Wonosari, 55861, Indonesia; 7 Faculty of Medicine, Universitas Brawijaya, Malang, East Java, 65117, Indonesia; 8 Department of Pharmacy, BGC Trust University Bangladesh, Chittagong-4381, Bangladesh; 9 Ragon Institute of MGH, MIT and Harvard, Cambridge, MA, 02139, USA; 10 Department of Microbiology, School of Medicine, Universitas Syiah Kuala, Banda Aceh, Aceh, 23111, Indonesia; 11 Molecular Diagnostic Laboratory, Johns Hopkins Aramco Healthcare, Dhahran, 31311, Saudi Arabia; 12 Division of Pathology, ICAR-Indian Veterinary Research Institute, Izatnagar, Bareilly, Uttar Pradesh, 243122, India; 13 Tropical Disease Centre, School of Medicine, Universitas Syiah Kuala, Banda Aceh, Aceh, 23111, Indonesia

**Keywords:** anosmia, COVID-19, dysgeusia, predictor, SARS-CoV-2

## Abstract

**Background**: The present study aimed to determine the global prevalence of anosmia and dysgeusia in coronavirus disease 2019 (COVID-19) patients and to assess their association with severity and mortality of COVID-19. Moreover, this study aimed to discuss the possible pathobiological mechanisms of anosmia and dysgeusia in COVID-19.

**Methods**: Available articles from PubMed, Scopus, Web of Science, and preprint databases (MedRxiv, BioRxiv, and Researchsquare) were searched on November 10th, 2020. Data on the characteristics of the study (anosmia, dysgeusia, and COVID-19) were extracted following the Preferred Reporting Items for Systematic Reviews and Meta-Analyses (PRISMA) guideline. Newcastle–Ottawa scale was used to assess research quality. Moreover, the pooled prevalence of anosmia and dysgeusia were calculated, and the association between anosmia and dysgeusia in presence of severe acute respiratory syndrome coronavirus 2 (SARS-CoV-2) was assessed using the Z test.

**Results**: Out of 32,142 COVID-19 patients from 107 studies, anosmia was reported in 12,038 patients with a prevalence of 38.2% (95% CI: 36.5%, 47.2%); whereas, dysgeusia was reported in 11,337 patients out of 30,901 COVID-19 patients from 101 studies, with prevalence of 36.6% (95% CI: 35.2%, 45.2%), worldwide. Furthermore, the prevalence of anosmia was 10.2-fold higher (OR: 10.21; 95% CI: 6.53, 15.96, *p* < 0.001) and that of dysgeusia was 8.6-fold higher (OR: 8.61; 95% CI: 5.26, 14.11, *p* < 0.001) in COVID-19 patients compared to those with other respiratory infections or COVID-19 like illness. To date, no study has assessed the association of anosmia and dysgeusia with severity and mortality of COVID-19.

**Conclusion**: Anosmia and dysgeusia are prevalent in COVID-19 patients compared to those with the other non-COVID-19 respiratory infections. Several possible mechanisms have been hypothesized; however, future studies are warranted to elucidate the definitive mechanisms of anosmia and dysgeusia in COVID-19.

**Protocol registration: **PROSPERO CRD42020223204. ****

## Introduction

Coronavirus disease 2019 (COVID-19), caused by severe acute respiratory syndrome coronavirus 2 (SARS-CoV-2), was initially identified in late December 2019 in Wuhan, Hubei Province, Republic of China ^1, 2^. This viral pandemic rapidly spread worldwide, infecting more than 60 million people, causing more than 1 million deaths ^3^, and severely affecting the global healthcare system ^4, 5^. Several drugs have been repurposed for treating COVID-19 ^5- 9^; however, no drug has been recommended or approved by the World Health Organization (WHO). The common symptoms of COVID-19 include dry cough, fever, dyspnea, fatigue, anorexia, diarrhea, chest pain, headache, and muscle ache ^10, 11^. In particular, two manifestations have been increasingly identified among asymptomatic people that later tested positive for the presence of SARS-CoV-2: anosmia and dysgeusia ^12^. Remarkably, previous studies reported that these olfactory issues were reported in 11.8% of COVID-19 cases before other symptoms occured ^13- 15^.

Anosmia, a severe condition of hyposmia, is a part of olfactory dysfunction where the person is unable to sense smell or detect odor ^16^. Dysgeusia is a sensory dysfunction where the individual loses the perception of taste ^17^. The British Association of Otorhinolaryngology reported that both dysfunctions varied from 3-20% among COVID-19 patients ^18^. A previous study among 42 patients revealed that more than a third presented anosmia and dysgeusia ^19^. A higher percentage of anosmia and dysgeusia cases were also reported ^20^. Furthermore, another study reported that anosmia in COVID-19 is related to the enlargement of bilateral olfactory bulb edema ^21^.

This evidence may be crucial in the present COVID-19 pandemic. As the real-time reverse transcriptase polymerase chain reaction (RT-PCR) test has certain limitations for screening, the manifestation of anosmia and dysgeusia could be used as an early warning for practitioners or clinicians to build a rationale to reach a firm conclusion on patients with SARS-CoV-2 infection ^22, 23^. Additionally, a recent study reported that anosmia and dysgeusia are among the earliest symptoms observed in COVID-19 patients ^24^; however, in-depth analysis of this dysfunction and its relation to the pathogenesis, severity, and mortality of COVID-19 is missing from the literature. Thus, the present study aimed to summarize the global evidence of anosmia and dysgeusia among COVID-19 patients, in order to assess their association with the severity and mortality of the disease, and provide a comprehensive review related to the possible pathogenesis of anosmia and dysgeusia in SARS-CoV-2 infection.

## Methods

### Registration and protocol

To comprehensively calculate the cumulative prevalence of anosmia and dysgeusia in SARS-CoV-2 infection worldwide, a systematic review was conducted following guideline of Preferred Reporting Items for Systematic Reviews and Meta-Analyses (PRISMA) ^25^. The protocol of this systematic review has been registered at PROSPERO ( CRD42020223204).

### Eligibility criteria of studies

All articles reporting anosmia and dysgeusia as the symptom of COVID-19 were included. COVID-19 case was defined by a positive RT-PCR for SARS-CoV-2 from either nasopharyngeal swab, oropharyngeal swab, bronchoalveolar lavage, or cerebrospinal fluid. All cross-sectional, retrospective, and prospective studies that randomly sampled COVID-19 cases from community or hospitals were considered eligible; whereas case reports and case series, including all editorials, reviews, and commentaries, were excluded. Studies targeting specific groups such as pregnant females, children, and other groups, were excluded. Only articles written in English during 2019-2020 were included.

### Information sources and search strategy

Three bibliographical databases (PubMed, Scopus, and Web of Science) and three preprint databases (MedRxiv, BioRxiv, and Researchsquare) were used to identify the potential articles (as of November 10th, 2020). The search criteria were as follows. PubMed ([Title] “SARS-CoV-2” OR “COVID-19” OR “Wuhan coronavirus” OR “Wuhan virus” OR “novel coronavirus” OR “nCoV” OR “severe acute respiratory syndrome coronavirus 2” OR “coronavirus disease 2019 virus” OR “2019-nCoV” OR “2019 novel coronavirus” OR “severe acute respiratory syndrome coronavirus 2” OR “coronavirus” OR “coronaviruses” OR “SARS 2” OR “2019-nCoV acute respiratory disease” OR “novel coronavirus pneumonia” OR “COVID”) AND ([All] “Anosmia” OR “smell loss” OR “smell dysfunction” OR “smell impairment” OR “hyposmia” OR “dysosmia” OR “olfactory dysfunction” OR “olfactory disorder”) AND (“dysgeusia” OR “taste loss” OR “taste dysfunction” OR “taste impairment” OR “gustatory dysfunction” OR “gustatory disorder” OR “hypogeusia” OR “ageusia”). Scopus ([Title] “SARS-CoV-2” OR “COVID-19” OR “Wuhan coronavirus” OR “Wuhan virus” OR “novel coronavirus” OR “nCoV” OR “severe acute respiratory syndrome coronavirus 2” OR “coronavirus disease 2019 virus” OR “2019-nCoV” OR “2019 novel coronavirus” OR “severe acute respiratory syndrome coronavirus 2” OR “coronavirus” OR “coronaviruses” OR “SARS 2” OR “2019-nCoV acute respiratory disease” OR “novel coronavirus pneumonia” OR “COVID”) AND ([All] “Anosmia” OR “smell loss” OR “smell dysfunction” OR “smell impairment” OR “hyposmia” OR “dysosmia” OR “olfactory dysfunction” OR “olfactory disorder”) AND (“dysgeusia” OR “taste loss” OR “taste dysfunction” OR “taste impairment” OR “gustatory dysfunction” OR “gustatory disorder” OR “hypogeusia” OR “ageusia”). Web of Science ([Title] “SARS-CoV-2” OR “COVID-19” OR “Wuhan coronavirus” OR “Wuhan virus” OR “novel coronavirus” OR “nCoV” OR “severe acute respiratory syndrome coronavirus 2” OR “coronavirus disease 2019 virus” OR “2019-nCoV” OR “2019 novel coronavirus” OR “severe acute respiratory syndrome coronavirus 2” OR “coronavirus” OR “coronaviruses” OR “SARS 2” OR “2019-nCoV acute respiratory disease” OR “novel coronavirus pneumonia” OR “COVID”) AND ([All] “Anosmia” OR “smell loss” OR “smell dysfunction” OR “smell impairment” OR “hyposmia” OR “dysosmia” OR “olfactory dysfunction” OR “olfactory disorder”) AND ([All] “dysgeusia” OR “taste loss” OR “taste dysfunction” OR “taste impairment” OR “gustatory dysfunction” OR “gustatory disorder” OR “hypogeusia” OR “ageusia”).

Moreover, we searched the preprint servers MedRxiv, BioRxiv, and Researchsquare for non-peer-reviewed articles. Data were extracted from the articles as well as supplementary materials. Reference lists from the eligible articles were retrieved for further relevant studies.

### Study selection and data extraction

The information of identified articles was imported into EndNote X9 (Thompson Reuters, Philadelphia, PA, USA). Duplicates between databases were removed. To identify eligible studies, the retrieved articles were screened based on title and abstract. The potentially eligible studies were then fully reviewed by two authors (MF and JKF). After reviewing the full texts, the eligibility of each study was decided.

Information of study characteristics, study site, study design, number of patients with anosmia, number of patients with dysgeusia, and COVID-19 characteristics such as number of patients, severity, and outcome were collected.

### Outcomes

The primary outcomes were: (a) the global incidence of anosmia in COVID-19 patients; (b) the global incidence of dysgeusia in COVID-19 patients; (c) the association of anosmia with the severity of COVID-19; (d) the association of dysgeusia with the severity of COVID-19; (e) the association of anosmia with mortality of COVID-19; and (f) the association of dysgeusia with mortality of COVID-19. Moreover, this review was conducted to provide the possible pathogenesis of anosmia and dysgeusia in SARS-CoV-2 infection.

### Data synthesis

The cumulative prevalence rate of anosmia and dysgeusia was calculated for COVID-19 cases by dividing the number of COVID-19 cases with anosmia by the total number of COVID-19 cases with and without anosmia, and was expressed as a percentage (%) with 95% confidence intervals (95% CI). Pooled odds ratios (OR) and 95% CI were calculated to assess the association of anosmia and the occurrence of SARS-CoV-2 compared to non-SARS-CoV-2 respiratory infections. The same method was used for dysgeusia. The pooled OR and 95% CI were presented in a forest plot.

### Risk of bias assessment

Critical assessment was conducted for the study setting and diagnosis of SARS-CoV-2 to reduce the bias. The Newcastle-Ottawa scale (NOS) ^26^ was used as critical appraisals to assess the quality of eligible studies. Prior to analysis, gathered data from studies were evaluated for heterogeneity and potential publication bias.

### Statistical analysis

To assess the association between anosmia or dysgeusia and the presence of SARS-CoV-2, Z test was performed ( *p* < 0.05 was considered statistically significant). Q test was used to evaluate the heterogeneity among studies, and the data with heterogeneity was analyzed using a random effect model. The reporting and publication bias were assessed using Egger’s test and a funnel plot ( *p* < 0.05 was considered having potential for publication bias). The data were analyzed using Review Manager version 5.3 ^27^.

## Results

### Study eligibility results

**Figure 1.**
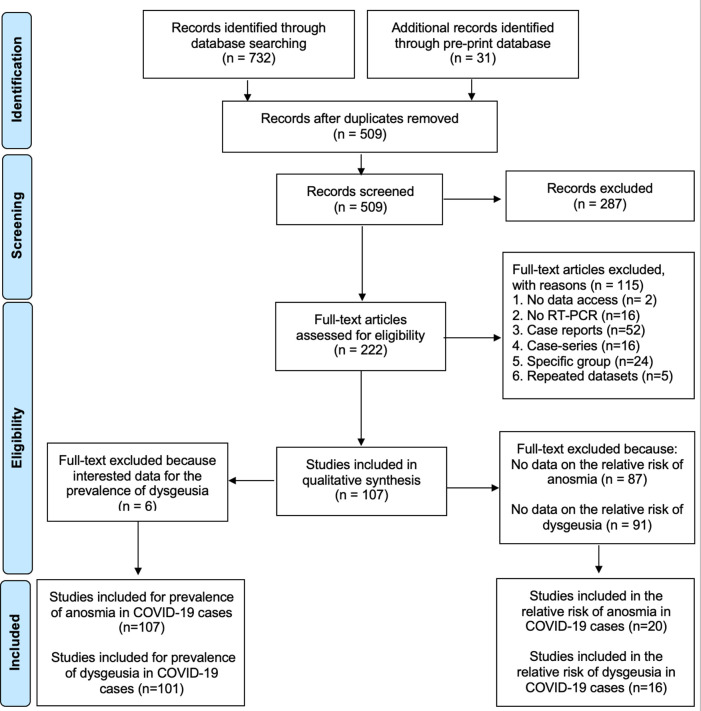
Flowchart of the result of literature search according to PRISMA.

In total, 691 articles (660 reviewed articles and 31 preprint articles) were identified through the databases; of these, 182 articles were removed as duplicates. An additional 287 articles were excluded following a screening process of the titles and abstracts due to irrelevant studies, leaving 222 references ( **Figure 1**). Full-texts of the remaining 222 references were retrieved and screened for eligibility, and this process excluded an additional 115 references as the inclusion criteria was not met. This exclusion included articles with no access ^28, 29^, RT-PCR not clearly stated in the text ^30- 45^, case reports ^46- 97^, case seriess ^98- 113^, repeated datasetss ^114- 118^, and studies in specific groups ^119- 130^. A complete assessment was conducted for 107 references.

The meta-analysis included 107 studies to calculate the prevalence of anosmia in COVID-19 patients. Additionally, 6 studies were excluded while calculating the prevalence of dysgeusia in COVID-19, thus leaving 101 eligible studies. In total, 20 and 16 studies were included to assess the association of anosmia and dysgeusia with the COVID-19 occurrence, respectively.

### The prevalence of anosmia and dysgeusia in COVID-19

To calculate the prevalence of anosmia in COVID-19 cases, 107 studies were included comprising 32,142 COVID-19 patients, and anosmia was reported in 12,038 patients with a global pooled prevalence of 38.2% (95% CI: 36.5%, 47.2%). The list of the studies and the prevalence of anosmia in each study are presented in **Table 1**.

In total, 30,901 COVID-19 patients from 101 studies were included to calculate the prevalence of dysgeusia in COVID-19. Dysgeusia was identified in 11,337 out of 30,901 COVID-19 patients resulting in a cumulative prevalence of 36.6% (95% CI: 35.2%, 45.2%). The individual studies and the prevalence of dysgeusia from each study are listed in **Table 2**.

**Table 1.** The prevalence of anosmia among COVID-19 patients around the globe.

Study Design	Country	COVID-19		Prevalence (%)	95%CI	Ref
		Anosmia	Total			
Retrospective	Singapore	53	305	17.38	13.12, 21.63	131
Prospective	Turkey	9	29	31.03	14.20, 47.87	119
Prospective	France	31	225	13.78	9.27, 18.28	132
Case control	Spain	25	79	31.65	21.39, 41.90	133
Retrospective	Taiwan	42	321	13.08	9.39, 16.77	134
Case control	Canada	69	134	51.49	43.03, 59.95	135
Case control	US	60	101	59.41	49.83, 68.98	136
Retrospective	Italy	13	213	6.10	2.89, 9.32	137
Cross sectional	US	40	59	67.80	55.87, 79.72	138
Cross sectional	Spain	138	197	70.05	63.65, 76.45	139
Cross sectional	Brazil	539	655	82.29	79.37, 85.21	140
Retrospective	Pakistan	4	30	13.33	1.17, 25.50	141
Retrospective	Spain	90	375	24.00	19.68, 28.32	142
Observational	Europe	997	1420	70.21	67.83, 72.59	143
Prospective	South Korea	68	172	39.53	32.23, 46.84	144
Prospective	France	62	197	31.47	24.99, 37.96	145
Retrospective	USA	45	251	17.93	13.18, 22.67	146
Retrospective	Italy	14	22	63.64	43.53, 83.74	147
Cross sectional	India	62	230	26.96	21.22, 32.69	148
Cross sectional	US	22	168	13.10	7.99, 18.20	149
Cross sectional	Hongkong	39	83	46.99	36.25, 57.73	150
Retrospective	France	54	114	47.37	38.20, 56.53	151
Retrospective	Japan	19	32	59.38	42.36, 76.39	152
Prospective	Taiwan	78	217	35.94	29.56, 42.33	153
Prospective	Turkey	18	172	10.47	5.89, 15.04	154
Retrospective	Italy	17	84	20.24	11.65, 28.83	155
Prospective	Italy	40	108	37.04	27.93, 46.14	156
Cross sectional	Egypt	80	96	83.33	75.88, 90.79	157
Retrospective	Kenya	279	787	35.45	32.11, 38.79	158
Cross sectional	Germany	29	73	39.73	28.50, 50.95	159
Prospective	UK	1	40	2.50	0.00, 7.34	160
Cross sectional	India	121	655	18.47	15.50, 21.45	161
Cross sectional	France	140	299	46.82	41.17, 52.48	162
Retrospective	France	54	114	47.37	38.20, 56.53	163
Prospective	Iran	22	92	23.91	15.20, 32.63	164
Retrospective	US	58	509	11.39	8.63, 14.16	165
Cross sectional	Spain	28	45	62.22	48.06, 76.39	166
Cross sectional	Brazil	28	73	38.36	27.20, 49.51	167
Prospective	Turkey	157	262	59.92	53.99, 65.86	168
Retrospective	France	17	55	30.91	18.70, 43.12	169
Cross sectional	UK	344	579	59.41	55.41, 63.41	170
Retrospective	Somalia	24	60	40.00	27.60, 52.40	171
Prospective	US	18	42	42.86	27.89, 57.82	172
Prospective	Turkey	33	143	23.08	16.17, 29.98	173
Retrospective	China	11	214	5.14	2.18, 8.10	174
Retrospective	Brazil	8	1208	0.66	0.20, 1.12	175
Retrospective	US	3	50	6.00	0.00, 12.58	176
Cross sectional	India	26	391	6.65	4.18, 9.12	177
Retrospective	China	34	86	39.53	29.20, 49.87	178
Retrospective	France	37	70	52.86	41.16, 64.55	179
Cross sectional	Italy	34	54	62.96	50.08, 75.84	180
Retrospective	UK	80	141	56.74	48.56, 64.92	181
Prospective	Italy	44	72	61.11	49.85, 72.37	182
Retrospective	Belgium	27	47	57.45	43.31, 71.58	183
Case control	Israel	3	16	18.75	0.00, 37.88	184
Case control	Turkey	50	81	61.73	51.14, 72.31	185
Retrospective	China, France Germany	154	394	39.09	34.27, 43.90	186
Retrospective	Malaysia	31	145	21.38	14.71, 28.05	187
Retrospective	Europe	357	417	85.61	82.24, 88.98	188
Retrospective	Italy	29	100	29.00	20.11, 37.89	189
Cohort	Italy	126	151	83.44	77.52, 89.37	190
Cross sectional	Switzerland	63	103	61.17	51.75, 70.58	191
Case control	Italy	26	43	60.47	45.85, 75.08	192
Retrospective	Germany	80	91	87.91	81.21, 94.61	193
Prospective	Israel	78	112	69.64	61.13, 78.16	194
Cohort	India	29	225	12.89	8.51, 17.27	195
Case control	Turkey	44	116	37.93	29.10, 46.76	196
Retrospective	Turkey	55	155	35.48	27.95, 43.02	197
Retrospective	France	1442	3737	38.59	37.03, 40.15	198
Retrospective	South Korea	5	328	1.52	0.20, 2.85	199
Prospective	US	23	46	50.00	35.55, 64.45	200
Cross sectional	Spain	46	58	79.31	68.89, 89.74	201
Cross sectional	Germany	22	34	64.71	48.64, 80.77	202
Cohort	US	145	273	53.11	47.19, 59.03	203
Retrospective	Europe	3	204	1.47	0.00, 3.12	204
Cohort	US	32	318	10.06	6.76, 13.37	205
Prospective	South Korea	389	3191	12.19	11.06, 13.33	206
Prospective	France	81	115	70.43	62.09, 78.78	207
Retrospective	China	30	196	15.31	10.27, 20.35	208
Retrospective	Iran	96	100	96.00	92.16, 99.84	209
Retrospective	Qatar	19	141	13.48	7.84, 19.11	210
Cross sectional		22	100	22.00	13.88, 30.12	211
Cross sectional	France	129	390	33.08	28.41, 37.75	212
Case control	India	11	74	14.86	6.76, 22.97	213
Retrospective	Turkey	529	1197	44.19	41.38, 47.01	214
Case control	Israel	76	112	67.86	59.21, 76.51	215
Cross sectional	Canada	31	56	55.36	42.34, 68.38	216
Retrospective	US	75	169	44.38	36.89, 51.87	217
Retrospective	China	134	1172	11.43	9.61, 13.26	218
Cohort	US	15	177	8.47	4.37, 12.58	219
Cross sectional	Greece	29	79	36.71	26.08, 47.34	220
Cross sectional	Saudi Arabia	28	128	21.88	14.71, 29.04	221
Cross sectional	Italy	283	508	55.71	51.39, 60.03	222
Cross sectional	Italy	237	355	66.76	61.86, 71.66	223
Cross sectional	Spain	26	31	83.87	70.92, 96.82	224
Cross sectional	Spain	454	846	53.66	50.30, 57.02	225
Prospective	Italy	84	138	60.87	52.73, 69.01	226
Case control	Brazil	23	57	40.35	27.61, 53.09	227
Case control	Iran	59	60	98.33	95.09, 100.00	228
Retrospective	US	198	949	20.86	18.28, 23.45	229
Prospective	Italy	46	50	92.00	84.48, 99.52	230
Cross sectional	Turkey	71	223	31.84	25.72, 37.95	231
Prospective	Italy	44	67	65.67	54.30, 77.04	232
Prospective	India	62	76	81.58	72.86, 90.29	233
Cross sectional	Brazil	159	179	88.83	84.21, 93.44	234
Retrospective	Global	1324	1698	77.97	76.00, 79.95	235
Retrospective	Italy	46	111	41.44	32.28, 50.61	236

**Table 2.** The prevalence of dysgeusia among COVID-19 patients around the globe.

Study design	Country	COVID-19		Prevalence (%)	95%CI	Ref
		Dysgeusia	Total			
Retrospective	Singapore	53	305	17.38	13.12, 21.63	131
Prospective	Turkey	6	29	20.69	5.95, 35.43	119
Case control	Spain	29	79	36.71	26.08, 47.34	133
Retrospective	Taiwan	42	321	13.08	9.39, 16.77	134
Case control	Canada	69	134	51.49	43.03, 59.95	135
Case control	US	60	101	59.41	49.83, 68.98	136
Retrospective	Italy	6	213	2.82	0.59, 5.04	137
Cross sectional	US	42	59	71.19	59.63, 82.74	138
Cross sectional	Spain	128	197	64.97	58.31, 71.64	139
Prospective	Brazil	502	655	76.64	73.40, 79.88	140
Retrospective	Pakistan	4	30	13.33	1.17, 25.50	141
Retrospective	Spain	90	375	24.00	19.68, 28.32	142
Cross sectional	Europe	770	1420	54.23	51.63, 56.82	143
Prospective	South Korea	58	172	33.72	26.66, 40.79	144
Prospective	France	56	197	28.43	22.13, 34.73	145
Retrospective	USA	41	251	16.33	11.76, 20.91	146
Retrospective	Italy	14	22	63.64	43.53. 83.74	147
Cross sectional	India	25	230	10.87	6.85. 14.89	148
Cross sectional	US	15	168	8.93	4.62, 13.24	149
Cross sectional	Hongkong	36	83	43.37	32.71, 54.04	150
Retrospective	France	54	114	47.37	38.20. 56.53	151
Retrospective	Japan	18	32	56.25	39.06, 73.44	152
Prospective	Taiwan	78	217	35.94	29.56, 42.33	153
Prospective	Turkey	11	172	6.40	2.74, 10.05	154
Retrospective	Italy	26	84	30.95	21.07, 40.84	155
Prospective	Italy	66	108	61.11	51.92, 70.31	156
Prospective	Iran	66	76	86.84	79.24, 94.44	122
Retrospective	Kenya	279	787	35.45	32.11, 39.79	158
Cross sectional	Germany	29	73	39.73	28.50, 50.95	159
Cross sectional	France	124	299	41.47	35.89, 47.05	162
Retrospective	France	46	54	85.19	75.71, 94.66	163
Prospective	Iran	15	92	16.30	8.76, 23.85	164
Retrospective	Illinois	81	509	15.91	12.74, 19.09	165
Cross sectional	Brazil	29	73	39.73	28.50, 50.95	167
Prospective	Turkey	157	262	59.92	53.99, 65.86	168
Retrospective	France	17	55	30.91	18.70, 43.12	169
Cross sectional	UK	344	579	59.41	55.41, 63.41	170
Retrospective	Somalia	17	60	28.33	16.93, 39.74	171
Prospective	US	24	42	57.14	42.18, 72.11	172
Prospective	Turkey	51	143	35.66	27.81, 43.52	173
Retrospective	China	12	214	5.61	2.52, 8.69	174
Retrospective	Brazil	3	1208	0.25	0.00, 0.53	175
Retrospective	Illinois	5	50	10.00	1.68, 18.32	176
Retrospective	China	12	214	5.61	2.52, 8.69	237
Cross sectional	India	35	391	8.95	6.12, 11.78	177
Retrospective	China	33	86	38.37	28.09, 48.65	178
Retrospective	France	34	70	48.57	36.86, 60.28	179
Cross sectional	Italy	34	54	62.96	50.08, 75.84	115
Retrospective	UK	89	141	63.12	55.16, 71.08	181
Prospective	Italy	39	72	54.17	42.66, 65.68	182
Case control	Turkey	43	52	82.69	72.41, 92.97	125
Prospective	Belgium	37	86	43.02	32.56, 53.49	238
Retrospective	Belgium	6	47	12.77	3.23, 22.31	183
Case control	Israel	3	16	18.75	0.00, 37.88	184
Case control	Turkey	22	81	27.16	17.47, 36.85	185
Retrospective	China, France Germany	100	394	25.38	21.08, 29.68	186
Retrospective	Malaysia	34	145	23.45	16.55, 30.34	187
Retrospective	Europe	342	417	82.01	78.33, 85.70	188
Retrospective	Italy	41	100	41.00	31.36, 50.64	189
Cohort	Italy	135	151	89.40	84.49, 94.31	190
Cross sectional	Switzerland	67	103	65.05	55.84, 74. 26	191
Prospective	Israel	82	112	73.21	65.01, 81.42	194
Cohort	India	39	225	17.33	12.39, 22.28	195
Case control	turkey	48	116	41.38	32.42, 50.34	196
Retrospective	turkey	25	155	16.13	10.34, 21.92	197
Retrospective	France	1389	3737	37.17	35.62, 38.72	198
Cross sectional	Iran	37	49	75.51	63.47, 87.55	129
Prospective	NA	476	751	63.38	59.94, 66.83	239
Retrospective	South Korea	3	328	0.91	0.00, 19.94	199
Cross sectional	Spain	51	58	87.93	79.55, 96.31	201
Cohort	US	145	273	53.11	47.19, 59.03	203
Retrospective	Europe	3	204	1.47	0.00, 3.12	204
Cohort	US	24	318	7.55	4.64, 10.45	205
Prospective	South Korea	353	3191	11.06	9.97, 12.15	206
Prospective	France	81	115	70.43	62.09, 78.78	207
Retrospective	China	23	196	11.73	7.23, 16.24	208
Retrospective	Qatar	28	141	19.86	13.27, 26.44	210
Cross sectional	India	40	100	40.00	30.40, 49.60	211
Cross sectional	France	130	390	33.33	28.65, 38.01	212
Retrospective	Turkey	526	1197	43.94	41.13, 46.75	214
Case control	Israel	80	112	71.43	63.06, 79.80	215
Cross sectional	Canada	32	56	57.14	44.18, 70.10	216
Retrospective	US	70	169	41.42	33.99, 48.85	217
Retrospective	China	242	1172	20.65	18.33, 22.97	218
Cohort	US	15	177	8.47	4.37, 12.58	219
Cross sectional	Greece	22	79	27.85	17.96, 37.73	220
Cross sectional	Saudi Arabia	28	128	21.88	14.71, 29.04	221
Cross sectional	Italy	321	508	63.19	58.99, 67.38	222
Cross sectional	Italy	232	355	65.35	60.40, 70.30	223
Cross sectional	Spain	4	31	12.90	1.10, 24.70	224
Cross sectional	Spain	442	846	52.25	48.88, 55.61	225
Prospective	Italy	56	138	40.58	32.39, 48.77	226
Case control	Brazil	5	57	8.77	1.43, 16.12	227
Case control	Iran	14	60	23.33	12.63, 34.04	228
Prospective	Italy	35	50	70.00	57.30, 82.70	230
Cross sectional	Turkey	77	223	34.53	28.29, 40.77	231
Prospective	Italy	17	67	25.37	14.95, 35.79	232
Prospective	India	64	76	84.21	76.01, 92.41	233
Cross sectional	Brazil	159	179	88.83	84.21, 93.44	234
Retrospective	Global	1149	1687	68.11	65.89, 70.33	235
Retrospective	Italy	66	111	59.46	50.33, 68.59	236

### Association of anosmia and the occurrence of COVID-19

**Figure 2.**
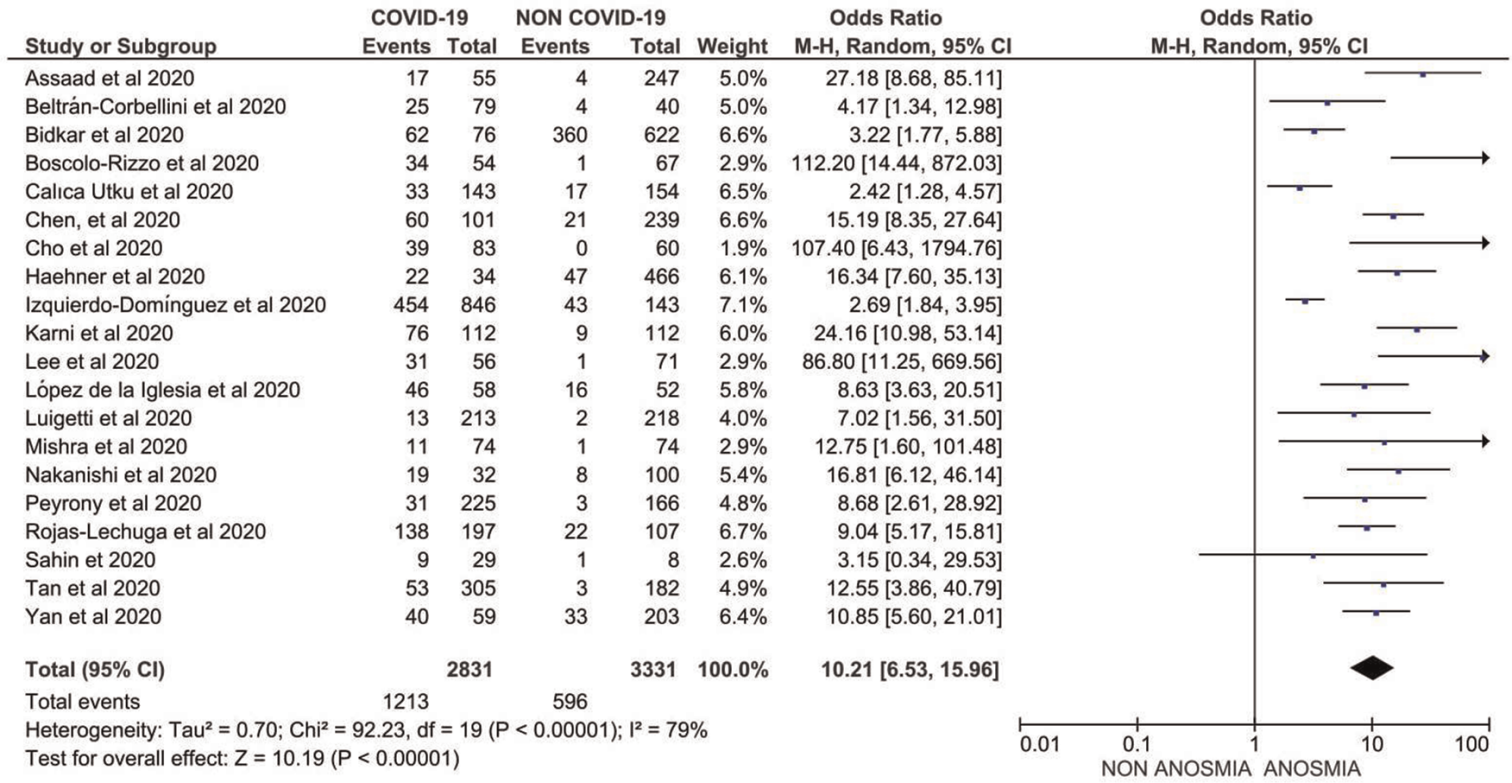
Forest plot of the association between anosmia and the risk of COVID-19 (OR: 10.21; 95%CI: 6.53, 15.96; p<0.001; Egger’ p=0.8340; heterogeneity p<0.001; I-squared 79.33%).

In total, 20 studies comprising 1,213 COVID-19 cases with anosmia and 2,735 non-COVID-19 patients (mostly COVID-19-like symptoms with negative RT-PCR for SARS-CoV-2) were analyzed to investigate the association between anosmia and the occurrence of COVID-19. Data suggested that anosmia was 10.2-fold more prevalent in patients with COVID-19 compared to those with COVID-19 like illness, OR 10.21 (95% CI: 6.53, 15.96) with *p* < 0.001 ( **Figure 2**).

### Association of dysgeusia and the occurrence of COVID-19

**Figure 3.**
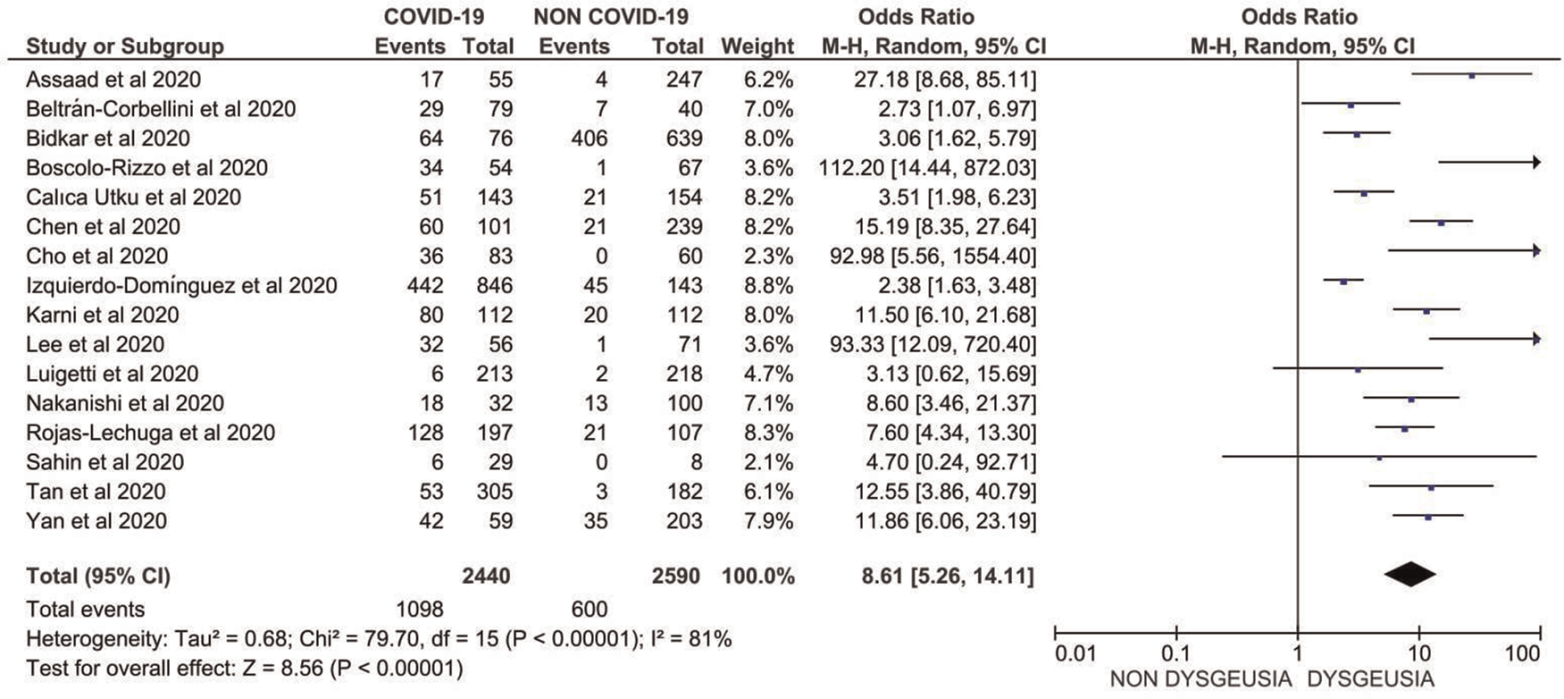
Forest plot of the association between dysgeusia and the risk of COVID-19 (OR: 8.61; 95%CI: 5.26, 14.11; p<0.001; Egger’ p=0.8220; heterogeneity p<0.001; I-squared 81.03%).

In total, 16 studies comprising 1,342 COVID-19 cases with dysgeusia and 1,990 patients with other respiratory illness (COVID-19 like illness with negative RT-PCR for SARS-CoV-2) were included to assess the association between dysgeusia and the occurrence of COVID-19. Data suggested that dysgeusia was 8.6-fold more prevalent in patients with COVID-19 compared to those with other respiratory illness, with OR 8.61 (95% CI: 5.26, 14.11) and *p*<0.001 ( **Figure 3** **)**.

### Association of anosmia and dysgeusia with COVID-19 severity and mortality

Limited studies have assessed the association between anosmia and dysgeusia and the severity and mortality of COVID-19 cases. One study linked anosmia with a lower fatality rate and a lower ICU admission ^240^.

## Discussion

### Anosmia and dysgeusia in COVID-19 patients

The pooled prevalence of anosmia in our systematic review was 38.2% of 32,142 COVID-19 cases. This result was almost thrice the initial prevalence reported from Wuhan, China ^174, 208^. This suggests that anosmia is a potential indicator of SARS-CoV-2 infection, and may be useful for screening and early identification of COIVD-19 patients, particularly asymptomatics ^241^. Some countries, such as the UK and US have used anosmia as an indicator for preventive measure, wherein COVID-19 patient with anosmia should commence self-isolation ^242- 244^.

Anosmia is not only present in COVID-19 patients, but also in patients with other respiratory diseases such as influenza, parainfluenza, Eipstein Barr virus, picornavirus, and rhinovirus ^245- 248^. However, our study demonstrated that the prevalence of anosmia was 10.2-fold higher in COVID-19 patient than that in non-COVID-19 patient. During the previous pandemics, such as severe acute respiratory syndrome (SARS) and Middle East respiratory syndrome (MERS), anosmia was rarely reported ^249^. Only one study reported persistent anosmia after 2 years of recovery from SARS ^250^. Another study reported that anosmia in COVID-19 patients varied based on ethnicity; anosmia in Caucasian is three times more prevalent than in Asian population ^251^.

Dysgeusia was initially reported in 11.7% of patients who were discharged from Wuhan hospital, which persisted for at least four weeks. This result was lower than ours (36.6% out of 30,901 COVID-19 cases), which might be attributable to either lower dysgeusia prevalence in China or underestimation of this symptom itself ^208^. Moreover, the prevalence of dysgeusia in COVID-19 patients was 8.6-fold higher than that in non-COVID-19 like illness. Herpes zoster and HIV have also been linked to gustatory dysfunction ^252, 253^. Furthermore, another study reported that anosmia and dysgeusia have 82.5% predictive value for positive SARS-CoV-2 RT-PCR ^254^.

### Possible pathogenesis of anosmia in COVID-19

Several mechanisms have been proposed to explain the emergence of anosmia in COVID-19 patients.

*a. Obstruction in the nasal airway*

As several viral infections in the respiratory system display blockage of nasal airway or nasal congestion, this hypothesis was initially proposed. According to this mechanism, the interaction between the odorants and olfactory receptors is inhibited by certain obstructions, thereby impairing the subsequent smelling processes ^255^. This condition results in anosmia. The obstruction could be caused by nasal discharge or by inflammation occurring in the nasal cavity ^256^; however, this hypothesis can be presumably ruled out. Moreover, several studies reported that anosmia is more prevalent than nasal congestion in COVID-19 patients ^188, 256- 259^. Interestingly, the incidence of rhinorrhea and nasal obstruction in SARS-CoV-2 infection is lower than other coronaviruses such as SARS-CoV and MERS-CoV ^260^.

Furthermore, presumably, nasal obstruction is a secondary mechanism by which anosmia is induced in COVID-19 patients as the obstruction in viral infection typically occurs as a subsequent event after damage in the mucociliary system, thereby inhibiting the nasal discharge and leading to nasal obstruction. In certain viral infections, the mucociliary system operated by ciliated cells is impaired. A previous study reported that human coronavirus (HCoV) disrupted the nasal ciliated respiratory epithelium leading to impaired mucociliary escalator system ^261^.

*b. Damage in olfactory sensory neurons*

Smelling processes commence when the odorants bind to the olfactory sensory neurons (OSNs) in the olfactory epithelium located in the nasal cavity, which subsequently transmits this information through their axons to the olfactory bulb in the brain ^262^. According to this concept, a viral attack on the receptor neurons eventually creates disturbances in the sense of smell; however, this hypothesis remains under debate as several recent studies reported the absence of angiotensin-converting enzyme 2 (ACE2) and transmembrane protease serine 2 (TMPRSS), the key factors for the virus to enter the cell ^263^, in the OSNs ^264- 267^. These findings are supported by another study carried out by Bryche *et al*., who demonstrated that SARS-CoV-2 was not detected in the OSNs of hamsters ^266^.

Moreover, after comparing the duration between anosmia incidence in COVID-19 patients and the normal cellular regeneration process, this proposed mechanism should be reconsidered. Several studies reported that COVID-19-related anosmia disappeared within 1-2 weeks, whereas regeneration of dead OSNs requires more than 2 week time period ^188, 206, 255, 262, 268^. This discrepancy results in a temporary conclusion that COVID-19-related anosmia is not directly associated with the impairment of the OSNs.

*c. Olfactory center damage in the brain*

The aforementioned dysfunction of OSNs and the mechanism by which SARS-CoV-2 directly affects the olfactory center via axonal transport of the neuron remains unclear, as the OSN lacks ACE2 and TMPRSS2 which hinders viral entry into the cell ^264- 267^. Nevertheless, the possibility of olfactory center disruption caused by SARS-CoV-2 should not be overlooked as the cause of anosmia, since a previous study concluded that human ACE2 (hACE2)-transgenic mice suffered from brain infection after intranasal inoculation with SARS-CoV ^269^. The study found that the brain infection commenced from the olfactory bulb, which is the axonal trajectory pathway of the OSNs ^269^. This finding suggests that SARS-CoV-2 might also first utilize another structure in the nasal cavity before it is transported into the OSNs.

*d. Olfactory supporting cells dysfunction*

As OSN does not express ACE2 and TMPRSS2, the virus should use another pathway to infect the olfactory system. Numerous studies have established the expression of these SARS-CoV-2 entry proteins in several supporting cells in olfactory epithelium, that is, Bowman’s gland cells, horizontal basal cells, olfactory bulb pericytes, mitral cells, sustentacular cells, and microvillar cells ^264- 267^. Of these supporting cells, the sustentacular cells have gained immense attention as the initial site of SARS-CoV-2 infection in the olfactory epithelium. In addition to their higher expression of ACE2 and TMPRSS2 than the others, sustentacular cells are located on the surface of the nasal cavity making them vulnerable to exposure to the external environment ^264, 267^.

Notably, sustentacular cells act as supporting cells and promote olfactory neuron in the olfactory system. These cells detoxify harmful odorants, promote odorant-receptor binding, and provide nutritional substances to support the action of olfactory receptor neurons ^255, 264^. Considerably, it is plausible to suggest that any damage occurring in sustentacular cells will in turn affect the olfactory epithelium and produce anosmia.

The corresponding regeneration time to the recovery of anosmia also supports the notion that sustentacular cell damage relates to anosmia caused by SARS-CoV-2. As the replenishment of dead OSNs does not correspond to the duration of COVID-19-related anosmia within 1-2 weeks, the regeneration of sustentacular cells seems to be in line with that time frame ^264, 266, 268^.

Furthermore, this hypothesis is supported by a recent study conducted by Bryche et al., who reported that SARS-CoV-2 was accumulated in sustentacular cells but not in the OSNs ^266^. The olfactory epithelial damage and sustentacular cell loss occurred 2 days after instilling SARS-CoV-2 intranasally in golden Syrian hamsters ^266^.

*e. Inflammation-related olfactory epithelium dysfunction*

It is worth noting that the cytokine storm in COVID-19 is strongly associated with organ dysfunctions, including OSNs ^232^. The dysfunction in this structure can lead to disturbance in the sense of smell ^270^. Torabi *et al*. suggested that proinflammatory cytokines, particularly tumor necrosis factor a (TNF-α), may lead to COVID-19-induced anosmia ^271^. Another proinflammatory cytokine, interleukin-6 (IL-6), increased in cases presenting with anosmia ^232^^,^^272^.

The mechanism used by these cytokines, in particular IL-6, to produce anosmia is not fully understood. Cazzolla *et al*. suggested that this effect can be caused by either peripheral or central action of the cytokines ^232^. In the periphery, IL-6 may induce apoptosis of ciliary neuronal cells in the olfactory epithelium ^272^, whereas in its central action, the olfactory center in the brain is attacked by the cytokine as a result of virus infection ^232^.

### Possible pathogenesis of dysgeusia in COVID-19

Although gustatory impairment is always displayed concomitantly with olfactory dysfunction, this symptom has a relatively different mechanism and is often distantly linked to the latter symptom. Several hypotheses have been proposed to explain the mechanism behind the emergence of dysgeusia in COVID-19 patients.

*a. The subsequent effect of cranial nerves dysfunction*

Considering the close relationship between the olfactory and gustatory system both peripherally and centrally, smell and taste dysfunction in COVID-19 often occurs concomitantly ^256, 273^. This hypothesis describes dysgeusia as a secondary event of olfactory dysfunction ^274^; however, several studies revealed that the percentage of dysgeusia in COVID-19 patients is higher than symptoms related to olfactory dysfunction ^188, 275^. Based on this finding, another mechanism may be involved in inducing SARS-CoV-2-related dysgeusia. Furthermore, COVID-19-induced dysgeusia could also occur when there is certain damage in the cranial nerves responsible for gustatory transmission (cranial nerve VII, IX, and X) ^276^. Among these nerves, SARS-CoV-2 exposure to cranial nerve VII has gained immense attention. Based on this hypothesis, the virus initially colonizes the nasopharynx structure, then moves to the Eustachian tube, and eventually reaches the middle ear where the virus gets access to chorda tympani and causes dysgeusia ^276^.

*b. Zinc deficiency*

Another interesting hypothesis underlying dysgeusia in COVID-19 is related to zinc deficiency ^276^. This hypothesis was developed as zinc is an important mineral in carbonic anhydrase, which is pivotal in maintaining taste sensation ^277^. Interestingly, one study reported that zinc level in patients with SARS-CoV-2 infection was significantly lower compared to that in the healthy control groups ^278^. Alterations in the sense of taste after being treated with certain treatments, such as irradiation in cancer patients ^279, 280^, could be prevented by zinc supplementation. Moreover, dengue fever virus and human immunodeficiency virus replication could be inhibited by zinc chelation ^281, 282^. Furthermore, pharmacological agents influencing ACE2 activity are associated with taste disturbances ^283, 284^.

Nevertheless, this effect does not relate to zinc deficiency as these drugs do not influence both serum and salivary zinc concentrations ^284^. Further investigation needs to be carried out to reveal the role of zinc in dysgeusia associated with COVID-19.

*c. SARS-CoV-2-bound sialic acid*

SARS-CoV-2 may produce dysgeusia via interaction with sialic acid receptors ^232, 274, 285^. Sialic acid plays a pivotal role in the taste processing pathway as it is a component of the normal salivary composition ^286^. Moreover, reduced amount of sialic acid impairs the ability to taste ^287^. An *in silico* study revealed that SARS-CoV-2 could interact with the sialic acid receptor through its spike protein ^288^. Previously, MERS-CoV was also reported to interact with this receptor ^289^. Following this occupancy, the gustatory threshold increases, while gustatory particles degrade at a higher rate ^274, 287^.

*d. Direct attack on several oral sites*

A previous study investigated the expression of ACE2 in various tissues in the oral cavity and found that the tongue had higher ACE2 expression in comparison to other tissues, such as buccal and gingival tissues ^290^. This finding raised a hypothesis that SARS-CoV-2 could directly attack the taste buds in the tongue, initiating inflammatory responses, and would eventually alter the sense of taste ^276^. It is proposed that the Toll-like receptor-mediated cascade and apoptosis are the subsequent events that could lead to taste dysfunction ^276, 291^.

A previous study investigating SARS-CoV infection in rhesus macaques revealed that, initially, the salivary gland was attacked by the virus ^292^. As the human salivary gland expresses a high level of ACE2 ^293^, it is reasonable to pay more attention to the vulnerability of this gland against SARS-CoV-2 exposure. Disruption in the activity of the salivary gland would produce either imbalance in salivary composition or impairment of salivary flow, which could ultimately result in dysgeusia ^276^.

## Conclusions

Out of 32,142 and 30,901 COVID-19 cases studied for anosmia and dysgeusia, respectively, the prevalence of anosmia was approximately 38.2%, whereas that of dysgeusia was 36.6%. Both of these symptoms were more common in COVID-19 compared to other respiratory infections (approximately 10 and 9 times, respectively). Several mechanisms have been proposed to explain the emergence of anosmia in COVID-19 patients including nasal airway obstruction, damage in OSNs, olfactory center damage in the brain, dysfunction of olfactory supporting cells, and inflammation-related olfactory epithelium dysfunction. Furthermore, some possible pathogenesis of dysgeusia in SARS-CoV-2 infection has been proposed including cranial nerve dysfunction, zinc deficiency, virion interaction, and direct attack of the virus to several oral sites.

## Data availability

### Undelying data

All data underlying the results are available as part of the article and no additional source data are required.

### Reporting guidelines

Figshare: PRISMA checklist for ‘Anosmia and dysgeusia in SARS-CoV-2 infection: Incidence, effects on COVID-19 severity and mortality, and the possible pathobiology mechanisms - A systematic review and meta-analysis’, https://doi.org/10.6084/m9.figshare.13323080.v1 ^294^.

Data are available under the terms of the Creative Commons Attribution 4.0 International license (CC-BY 4.0).
