# Supplementary material for: Anosmia and dysgeusia in SARS-CoV-2 infection: incidence and effects on COVID-19 severity and mortality, and the possible pathobiology mechanisms - a systematic review and meta-analysis
Source: F1000Res. 2021 Jan 21;10:40. [Version 1] doi: 10.12688/f1000research.28393.1 (PMC7993408; doi:10.12688/f1000research.28393.1)
Supplement: Peer review history

# Peer review: Reviewer response for version 1

**DOI:** 10.5256/f1000research.31412.r80213

**Published:** 2021-03-23

**Authors:** Mahir Gachabayov

**Affiliations:** 1 Department of Surgery, New York Medical College, Valhalla, NY, USA

Thank you for an interesting systematic review.

 This systematic review and meta-analysis aimed at evaluating the rate of anosmia and dysgeusia in patients with confirmed COVID-19 and their association with disease severity and mortality. 

 The research question makes sense and the methodology chosen for statistical analysis is logical. 

 The Introduction is comprehensible and easy to read, it clearly states the gap in the literature and the rationale behind this research question. The aim of the study was stated clearly.

 The Methods are well-formulated. Reporting of this review is compliant with the PRISMA guidelines. The search strategy is comprehensive, includes major databases as well as preprint servers. The details of the search strategy and the PRISMA flow diagrams were provided. Eligibility criteria and definitions were reported. Statistical analysis was adequate.

 The Results are well-written and clear. The findings of the statistical analysis were nicely summarized in tables and illustrated in forest plots. 

 The Discussion is comprehensible and easy to read. The authors have provided interpretation of their findings, and clinical and scientific implications thereof. The authors have provided possible etiopathogenesis for anosmia and dysgeusia in COVID-19 patients. 

 The Conclusion was justified by the statistical findings.

 In order to improve the manuscript, I have a few suggestions:

1. There is some confusion between the terms incidence and prevalence. In fact, in the Methods under the Outcomes, the authors stated the primary outcomes to be incidence of anosmia and dysgeusia, whereas they described this metrics as prevalence in the rest of the manuscript. I believe neither of the terms fits the context. I would change these terms to rate, eg. anosmia rate (rate of anosmia) and dysgeusia rate (rate of dysgeusia).
2. I would not use Newcastle-Ottawa scale for risk of bias assessment as it does not evaluate for heterogeneity due to the differences in the definitions of outcomes and interventions to measure the outcome. I would rather use ROBINS-I tool.
3. I would address in the Discussion the reasons for substantial heterogeneity in the pooled rates of anosmia and dysgeusia. I would consider heterogeneity in geographic locations and community types, heterogeneity in the definitions of anosmia and dysgeusia, and heterogeneity in the definitions of disease severity across the included studies.
4. I would add a brief paragraph to the Discussion (last paragraph) and acknowledge the strengths and limitations of this study.

Are the rationale for, and objectives of, the Systematic Review clearly stated?

Yes

Is the statistical analysis and its interpretation appropriate?

Yes

Are sufficient details of the methods and analysis provided to allow replication by others?

Yes

Are the conclusions drawn adequately supported by the results presented in the review?

Yes

Reviewer Expertise:

clinical outcomes and evidence synthesis

I confirm that I have read this submission and believe that I have an appropriate level of expertise to confirm that it is of an acceptable scientific standard.

# Peer review: Reviewer response for version 1

**DOI:** 10.5256/f1000research.31412.r80214

**Published:** 2021-03-22

**Authors:** Seyi Samson Enitan

**Affiliations:** 1 Department of Medical Laboratory Science, Babcock University, Ilishan-Remo, Nigeria

The present study examined the global prevalence of anosmia and dysgeusia in COVID-19 patients, their association with severity and mortality of COVID-19, as well as the possible pathobiological mechanisms of anosmia and dysgeusia in COVID-19. Authors reported a global prevalence of 38.2% and 36.6% for anosmia and dysgeusia, respectively. Identified potential mechanisms for anosmia include: Obstruction in the nasal airway, damage in olfactory sensory neurons, olfactory center damage in the brain, olfactory supporting cells dysfunction and inflammation-related olfactory epithelium dysfunction. On the other hand, the subsequent effect of cranial nerves dysfunction, Zinc deficiency, SARS-CoV-2-bound sialic acid, Direct attack on several oral sites are opined to be responsible for the dysgeusia.

 The work is okay and the findings are worth-sharing with the scientific community. The introduction is considered satisfactory. Authors provided background that puts the manuscript into context and allows readers outside the field to understand the purpose and significance of the study. They also identified the existing gap in knowledge that needs to be filled. The methodology section was clearly presented to allow the reproduction of the study. Discussion and Conclusion were well written. However, the limitation of the study was not clearly stated. And except, the association of anosmia and dysgeusia with severity and mortality is properly discussed in the study, it should be deleted from the objectives.

**Rating of the manuscript: **Use (1 = Excellent) (2 = Very Good) (3 = Average) (4 = Fair) (5 = poor)

 Originality

 2

 Contribution To The Field

 1

 Technical Quality

 2

 Clarity of Presentation

 2

 Depth of Research

 2

**Recommendation:  **Minor corrections are needed.

Are the rationale for, and objectives of, the Systematic Review clearly stated?

Yes

Is the statistical analysis and its interpretation appropriate?

Yes

Are sufficient details of the methods and analysis provided to allow replication by others?

Yes

Are the conclusions drawn adequately supported by the results presented in the review?

Yes

Reviewer Expertise:

Medical Virology and Immunology of Infectious Diseases.

I confirm that I have read this submission and believe that I have an appropriate level of expertise to confirm that it is of an acceptable scientific standard.

# Peer review: Reviewer response for version 1

**DOI:** 10.5256/f1000research.31412.r77883

**Published:** 2021-02-16

**Authors:** Cissy Kartasasmita

**Affiliations:** 1 Department of Child Health, Faculty of Medicine, Universitas Padjadjaran, Bandung, Indonesia

**Objectives of study**

*‘"The present study aimed to summarize the global evidence of anosmia and dysgeusia among COVID-19 patients, in order to assess their association with the severity and mortality of the disease, and provide a comprehensive review related to the possible pathogenesis of anosmia and dysgeusia in SARS-CoV-2 infection.”*

- The analysis on the assessment of the association with the severity and mortality of the disease is very short and needs more data to be reported.

**Association of anosmia and dysgeusia with COVID-19 severity and mortality**

*"Limited studies have assessed the association between anosmia and dysgeusia and the severity and mortality of COVID-19 cases. One study linked anosmia with a lower fatality rate and a lower ICU admission."*

- In the conclusion the authors did not include all the objectives of the study.
- No statement on the association anosmia and dysgeusia with severity and mortality as stated in the objectives.

**Conclusions**

- My recommendation would be to add the limitations of this study.

Are the rationale for, and objectives of, the Systematic Review clearly stated?

Yes

Is the statistical analysis and its interpretation appropriate?

Yes

Are sufficient details of the methods and analysis provided to allow replication by others?

Yes

Are the conclusions drawn adequately supported by the results presented in the review?

Partly

Reviewer Expertise:

Pediatrics, Epidemiology, Vaccinology, and Respirology

I confirm that I have read this submission and believe that I have an appropriate level of expertise to confirm that it is of an acceptable scientific standard, however I have significant reservations, as outlined above.
